# Antibiotics, Bacteria, and Antibiotic Resistance Genes: Aerial Transport from Cattle Feed Yards via Particulate Matter

**DOI:** 10.1289/ehp.1408555

**Published:** 2015-01-22

**Authors:** Andrew D. McEachran, Brett R. Blackwell, J. Delton Hanson, Kimberly J. Wooten, Gregory D. Mayer, Stephen B. Cox, Philip N. Smith

**Affiliations:** 1 Department of Environmental Toxicology, Texas Tech University, Lubbock, Texas, USA; 2 Research and Testing Laboratory, Lubbock, Texas, USA

## Abstract

**Background:**

Emergence and spread of antibiotic resistance has become a global health threat and is often linked with overuse and misuse of clinical and veterinary chemotherapeutic agents. Modern industrial-scale animal feeding operations rely extensively on veterinary pharmaceuticals, including antibiotics, to augment animal growth. Following excretion, antibiotics are transported through the environment via runoff, leaching, and land application of manure; however, airborne transport from feed yards has not been characterized.

**Objectives:**

The goal of this study was to determine the extent to which antibiotics, antibiotic resistance genes (ARG), and ruminant-associated microbes are aerially dispersed via particulate matter (PM) derived from large-scale beef cattle feed yards.

**Methods:**

PM was collected downwind and upwind of 10 beef cattle feed yards. After extraction from PM, five veterinary antibiotics were quantified via high-performance liquid chromatography with tandem mass spectrometry, ARG were quantified via targeted quantitative polymerase chain reaction, and microbial community diversity was analyzed via 16S rRNA amplification and sequencing.

**Results:**

Airborne PM derived from feed yards facilitated dispersal of several veterinary antibiotics, as well as microbial communities containing ARG. Concentrations of several antibiotics in airborne PM immediately downwind of feed yards ranged from 0.5 to 4.6 μg/g of PM. Microbial communities of PM collected downwind of feed yards were enriched with ruminant-associated taxa and were distinct when compared to upwind PM assemblages. Furthermore, genes encoding resistance to tetracycline antibiotics were significantly more abundant in PM collected downwind of feed yards as compared to upwind.

**Conclusions:**

Wind-dispersed PM from feed yards harbors antibiotics, bacteria, and ARGs.

**Citation:**

McEachran AD, Blackwell BR, Hanson JD, Wooten KJ, Mayer GD, Cox SB, Smith PN. 2015. Antibiotics, bacteria, and antibiotic resistance genes: aerial transport from cattle feed yards via particulate matter. Environ Health Perspect 123:337–343; http://dx.doi.org/10.1289/ehp.1408555

## Introduction

Bacterial resistance to antibiotics increasingly hinders treatment of life-threatening illnesses. Misuse and overuse of antibiotics plays a critical role in development of resistance and there is evidence that agricultural use of antibiotics is a contributor to the aggregation of resistance in the environment (Gilchrist et al. 2007; Levy and Marshall 2004). Nearly 10 million kilograms of antibiotics per year (likely an underestimation because of the lack of reporting requirements) are used in animal agriculture in the United States alone (Mellon et al. 2001; Sarmah et al. 2006). Antibiotics are administered to beef cattle to treat and prevent disease and to promote growth (Khan et al. 2008; Phillips et al. 2004; Shuford and Patel 2005). Antibiotics used for growth promotion are added to livestock feed, and after ingestion are incompletely metabolized and poorly absorbed in the gastrointestinal tract, resulting in excretion of parent compounds and metabolites (Boxall et al. 2006; Chee-Sanford et al. 2009; Khan et al. 2008; Shuford and Patel 2005; Wegener 2003). Upon excretion, these compounds may be transported into the environment beyond feed yard boundaries via application of manure waste onto agricultural fields, runoff, and, as reported here, airborne particulate matter (PM) (Chee-Sanford et al. 2009; Wegener 2003). Once in the environment, antibiotics can facilitate *de novo* development of bacterial antibiotic resistance and provide a selective advantage for bacteria that acquire resistance either in treated animals or in the environment (Chee-Sanford et al. 2009; Gilchrist et al. 2007; Silbergeld et al. 2008).

In 2012, there were 2,100 large-scale beef cattle feeding operations (> 1,000 head of cattle) in the United States [National Agricultural Statistics Service (NASS) 2013]. As of 1 March 2014, 76.3% of all cattle residing on U.S. feed yards with > 1,000 head (8.24 million cattle) were located in Texas, Oklahoma, Kansas, Nebraska, and Colorado (NASS 2014). Together, portions of these states constitute a region that has the highest frequency of dust storms in the United States (Orgill and Sehmel 1976) and the highest density of feed yards. Climatic conditions in this semi-arid region are conducive to wind scouring of dry soils, as well as aerial transport and deposition of PM onto surrounding soil surfaces, water surfaces, and vegetation and other living organisms. Moreover, cattle behavior facilitates daily suspension of PM above feed yards (see Supplemental Material, Figure S1). Relative humidity and soil moisture levels in this region are typically highest in the early morning hours and decline throughout the day. As a consequence, material on feed yard pen floors, which consists primarily of urine and fecal material, becomes dry and brittle, thus becoming source material for fugitive dust (Von Essen and Auvermann 2005). Despite management practices employed at many feed yards, such as landscaped windbreaks, frequent pen scraping, and sprinkling to mitigate dust production, cattle activity and movement in late afternoon and early evening result in pulverization and subsequent aerosolization of pen floor material. Wind speeds and temperatures are generally lowest early in the day, increasing throughout the afternoon, and decreasing again in the evening hours. Stable atmospheric conditions with minimal vertical mixing and turbulence also facilitate suspension of PM into air above feedlots. Fronts and other major weather patterns frequently sweep through this region and are often associated with exceedingly high wind velocities, which themselves transport significant masses of particulates into the atmosphere and across the region and continent (see Supplemental Material, Figure S2). Thus, in semi-arid regions where a majority of beef cattle feed yards exist in North America, transport of livestock-generated organic wastes occurs largely via wind. Under certain conditions, the Central Plains region of the United States becomes a large open source area producing dust on a scale commensurate with its vast size (Stout 2001).

People living in the vicinity of feed yards often complain about excessive dust, and airborne microorganisms and by-products from feed yards are considered potential human health threats (Dungan 2010). Yet the health of feed yard workers and nearby residents has not been thoroughly assessed (Von Essen and Auvermann 2005). There is increasing potential for human exposure to airborne dusts and associated antibiotics and microbes as population centers grow (Dungan 2010). Further, there appears to be consensus among numerous climate models that the American Southwest will continue to dry throughout the 21st century, potentially returning to Dust Bowl–era and 1950s drought conditions (Seager et al. 2007). Although extended periods of drought may result in reduced density of feed yards in affected regions, trends within the industry suggest that future consolidation of small feed yard operations into larger, corporate-owned operations will increase, particularly in arid regions where conditions are ideal for beef production (Galyean et al. 2011). If so, exposure to feed yard–derived dusts, antibiotics, and microbes will increase, not only via direct inhalation but also from deposition onto skin and food and into water (Dungan 2010).

Given the extent of confined beef cattle production, extensive use of veterinary antibiotics, and significant wind energy potentials in the Central Plains region of the United States, we hypothesized that antibiotics and antibiotic resistant bacteria in PM collected downwind of beef cattle feed yards would be more abundant than that in corresponding PM collected upwind. To address our hypothesis, we quantified antibiotics commonly administered in animal-based agriculture, assessed microbial community composition, and assessed occurrence of antibiotic resistance genes in airborne PM emanating from beef cattle feeding operations.

## Materials and Methods

*Sample collection*. Particulate matter samples were collected adjacent to 10 commercial beef cattle feed yards in the Southern High Plains. Feed yards included in this study had capacity of 20,000–50,000 head of cattle and were selected based on size and accessibility, including being within a 200-mi radius of Lubbock, Texas. Two feed yards (feed yards 7 and 8) sampled in this study were within 1 mi of each other but were not downwind of one another at the time of sampling. The remaining 8 feed yards in this study were not within 5 mi of another feed yard. PM was collected using Hi-Q CF-902 digital portable high-volume air samplers (HI-Q Environmental Products) fitted with 4-in. glass fiber filters to collect total suspended particulates (TSP) (see Supplemental Material, Figure S3). For analytical detection, samplers were placed 2–3 m above the ground approximately 10–20 m from feed yard boundaries, and samples were collected upwind and downwind of feed yards for 30 min at each location to obtain sufficient mass. Filter cartridges and holding containers were washed with 70% ethanol after each use. Sampling took place in the late afternoon between 1600 and 2000 hours, when cattle are most active and the greatest amount of PM is produced (Purdy et al. 2007). Sampling occurred between August and December 2012, and during weather conditions that were similar at all feed yards and characterized by moderate temperatures (mean = 18 ± 2°C), slight winds (mean = 3.0 ± 0.5 m/sec), low relative humidity, and no precipitation (see Supplemental Material, Table S4). All PM samples were transported to the laboratory on ice, weighed upon arrival to determine PM mass, and then frozen at –80°C until analysis.

*Quantitation of veterinary antibiotics in TSP*. Extraction and clean-up. Veterinary antibiotics have been previously identified and quantified using high-performance liquid chromatography with tandem mass spectrometry (LC-MS/MS) (Aust et al. 2008; Kim and Carlson 2007). PM samples were analyzed following the method of Kim and Carlson (2007) with minor adaptations. Briefly, entire filters were placed in 40-mL polypropylene tubes and twice extracted with 40 mL of ammonium hydroxide buffer solution (pH 10) or McIlvaine buffer solution (pH 4)—depending on the target compound—and 200 μL K_2_EDTA. Samples were filtered through syringes with DIONEX ASE cellulose filters (Dionex Corporation) before being loaded on to preconditioned Strata-X High Performance Polymeric solid phase extraction cartridges (Phenomenex) for cleanup. Cartridges were eluted with methanol, evaporated to dryness, and reconstituted in 1 mL of 10:90 methanol:water plus 0.1% formic acid.

LC-MS/MS parameters. LC-MS/MS using electrospray ionization (ESI) has been used for the identification and quantification of antibiotics in a variety of matrices (Hirsch et al. 1998; Kim and Carlson 2006, 2007; Luo and Ang 2000). Target compounds were separated via reverse-phase chromatography utilizing a 100 × 2.1-mm Kinetex PFP column (Phenomenex) with a 1.7-μm particle size and a Thermo Accela 1250 solvent pump (Thermo Fisher Scientific) equipped with a PAL autosampler (Agilent Technologies). Mobile-phase conditions were identical for all target compounds in the analysis (see Supplemental Material, Table S1). For identification and quantification of target compounds, we used MS/MS with ESI on a Thermo TSQ Quantum Access Max system (Thermo Scientific) in positive ion mode. Operating conditions for MS/MS were optimized for each compound, and the two most prevalent product ions were used for target compound identification and quantification (see Supplemental Material, Table S2).

*DNA extraction*. DNA was extracted from PM bound to filters using a PowerSoil DNA Isolation Kit (MoBio Laboratories) with minor adaptations. In lieu of soil, slices of each filter containing bound PM were taken from the center of the filters and added to the PowerSoil bead tube; PM that had become dislodged from filters while in storage was included in the PowerSoil bead tube for extraction. Extraction proceeded according to the kit directions, resulting in 100 μL of DNA in elution buffer (10 mM Tris). Concentrations of DNA in each sample were measured and recorded using a NanoDrop Spectrophotometer (Thermo Scientific) to account for total DNA used in quantitative polymerase chain reaction (qPCR).

*Real-time PCR assays for detection of resistant genes*. We used qPCR to detect the presence of antibiotic resistant genes using previously published primer sets for six tetracycline resistance genes (Peak et al. 2007; Smith et al. 2004) (see Supplemental Material, Table S3). Of the six tetracycline resistance genes targeted in this study, four (TetM, TetO, TetQ, and TetW) encode ribosomal protection proteins that remove tetracycline from the ribosome in a GTP-hydrolysis–dependent fashion, and two (TetB and TetL) code for cellular efflux proteins as their antibiotic resistant mechanisms (Peak et al. 2007). We included the selected resistance genes in this study on the basis of their previously reported detection in waste lagoons proximate to feed yard operations (Peak et al. 2007; Pei et al. 2006; Smith et al. 2004; Wu et al. 2010) and because of heavy veterinary use of tetracycline and sulfonamide antibiotics (Hamscher et al. 2002; Kemper 2008; Lindsey et al. 2001). qPCR analyses were performed on Lightcycler 480 PCR systems using LightCycler 480 DNA SYBR Green 1 Master Mix consisting of FastStart Taq DNA Polymerase, dNTP mix, and SYBR Green I dye (all from Roche). Temperature cycling was modeled after that of Pei et al. (2006), and entailed 1 cycle of denaturation at 95°C for 10 min, followed by amplification at 35 cycles of 15 sec at 95°C and 1 min at 60°C for all six gene-specific reactions. Because of the potential for erroneous amplicon production within the last 5 cycles of the reaction, target genes were considered present in the sample when amplified before 30 cycles of qPCR. We confirmed that amplicons produced through qPCR were single amplicons using agarose gel electrophoresis, followed by Sanger sequencing to verify amplification of the correct, unique DNA sequence. Melt curve analysis also verified single amplicon generation in each qPCR reaction. Amplicons in samples were quantified using a standard curve derived from amplification of known template amounts of synthesized oligonucleotide for each unique amplicon (Integrated DNA Technologies).

*Microbial diversity analysis*. The relationship between microbial communities present in PM upwind and downwind of feed yards was determined through DNA pyrosequencing. Samples were analyzed following 16S rRNA amplification and sequencing as described by Dowd et al. (2008), targeting the V1–V3 regions of the 16S rRNA gene. Analysis was performed at Research and Testing Laboratory.

Bioinformatics. Any sequence that contained a low-quality barcode or that failed to be at least half the expected amplicon length (or 250 bp, whichever was shortest) was removed from the data pool. Therefore, two samples were removed from analysis because of an insufficient number of quality sequencing reads. All sequences then were denoised using an algorithm based on USEARCH (Edgar 2010) and checked for chimeras using UCHIME (Edgar et al. 2011). The sequence data processing pipeline followed that outlined by Research and Testing Laboratory (2014). After denoising and chimera checking, sequence data were separated into operational taxonomic units (OTUs) and annotated using the RDP classifier (Wang et al. 2007) with GreenGenes v. 12.10 (McDonald et al. 2012) used as a reference. Finally, relative abundances of taxa at each hierarchical taxonomic level were calculated using the summarize_taxa.py QIIME script (http://www.qiime.org).

*Data analysis*. Microbial communities present in PM were analyzed to determine whether differences existed between downwind and upwind PM. The number of OTUs was used as a measure of bacterial species richness and was standardized to a consistent sampling effort using rarefaction prior to analysis. Differences in richness between upwind and downwind samples were evaluated using a paired *t*-test. Weighted UniFrac distances (Lozupone et al. 2006), which measure phylogenetic distances among samples, were used to characterize beta diversity among all samples, and were illustrated using Principal Coordinates Analysis (PCoA). Group differences (upwind vs. downwind) with respect to the overall microbiome were evaluated statistically using distance-based redundancy analysis (Anderson and Willis 2003). Within- and among-group patterns of beta diversity were examined using an analysis of variance–like permutation test available in the “vegan” package (vegan: Community Ecology, version 1.17–1; http://cran.r-project.org/web/packages/vegan/). To further categorize differences between upwind and downwind PM samples, paired *t*-tests were used to determine the statistical significance of differences in total PM mass, differences in the most abundant genera and phyla observed, and differences in antibiotic concentrations. UniFrac distances and rarefaction analysis were calculated using QIIME and all analyses were performed in R (version 2.14.1; http://cran.r-project.org/).

## Results

*PM and antibiotic concentrations*. The mass of PM collected immediately downwind of feed yards was significantly different from that collected immediately upwind of each feed yard (*p* = 0.002) (see Supplemental Material, Table S5). Utilizing both a high and low pH extraction buffer, we maximized extraction efficiency to the extent possible given the complex matrix of feed yard–derived PM. Monensin, a polyether ionophore antibiotic, was detected in 100% of PM samples downwind and upwind of feed yards, albeit below limits of quantitation in PM samples collected upwind of feed yards. Downwind of feed yards, the mean (± SE) monensin concentration was 1,800 ± 370 ng/g PM. Tylosin, a macrolide antibiotic, was detected in 80% of PM samples downwind of feed yards at 340 ± 92 ng/g PM, significantly lower than concentrations of monensin across all feed yards.

**Figure 1:**
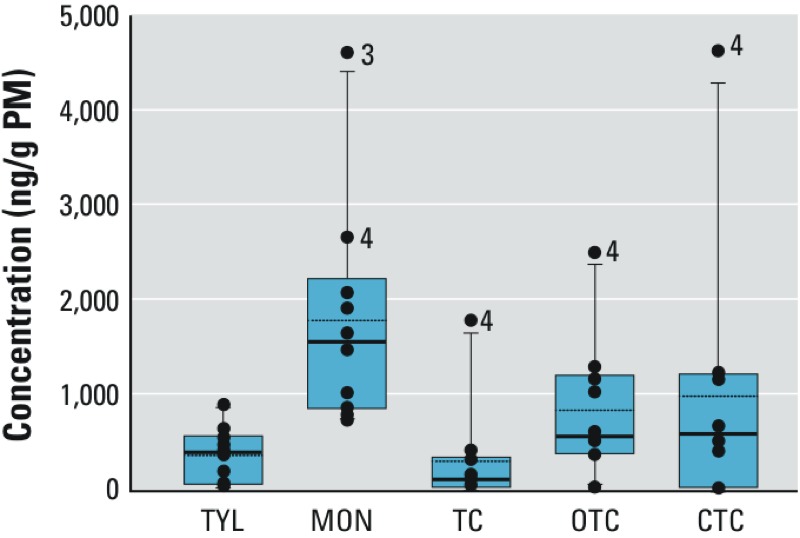
Concentrations (ng/g PM) of five targeted veterinary antibiotics measured in PM collected immediately downwind of feed yards (*n* = 10). Abbreviations: CTC, chlortetracycline; MON, monensin; OTC, oxytetracycline; TC, tetracycline; TYL, tylosin. Individual data points represent concentrations from each feed yard. Boxes extend from the 25th to the 75th percentile, solid horizontal lines represent the median of each distribution, and dotted horizontal lines represent the mean. Numbers within the graph indicate the specific feed yard. Feed yard 4 had consistently elevated concentrations of antibiotics.

Three tetracycline antibiotics (tetracycline, chlortetracycline, and oxytetracycline) were detected together in most PM samples collected downwind of feed yards (60%); oxytetracycline was the most frequently detected of the three and was detected in 100% of PM samples collected downwind of feed yards. Mean concentrations were 280 ± 170 ng/g PM tetracycline, 820 ± 220 ng/g oxytetracycline, and 970 ± 430 ng/g chlortetracycline. In addition, oxytetracycline was detected in 30% of upwind samples at concentrations below the limit of quantitation. Overall, monensin was present at the highest concentrations in downwind PM, followed by chlortetracycline and oxytetracycline (Figure 1). One particular site, feed yard 4, had consistently elevated concentrations of all antibiotics compared with other feed yards.

*Bacterial community structure analysis*. On the basis of amplicon sequencing of the V1–V3 variable regions of ribosomal RNA genes, we observed a total of 8,986 16S OTUs across all samples. The number of PM-associated OTUs was significantly higher among samples from downwind of feed yards compared with those collected upwind (*p* = 0.0095), indicating greater diversity in downwind PM (see Supplemental Material, Figure S4). In addition, bacterial phyla and genera common to fecal matter and gut flora were significantly more abundant in downwind PM samples compared with upwind PM samples (see Supplemental Material, Figures S5 and S6). Within these PM-associated bacterial communities, there were several genera that contain subtaxa known to be infectious in humans, such as *Corynebacterium* (present in 90% of all samples, including 100% of downwind and 80% of upwind samples), *Leptospira*, *Clostridium*, *Bacteroides*, and *Staphylococcus*.

**Figure 2:**
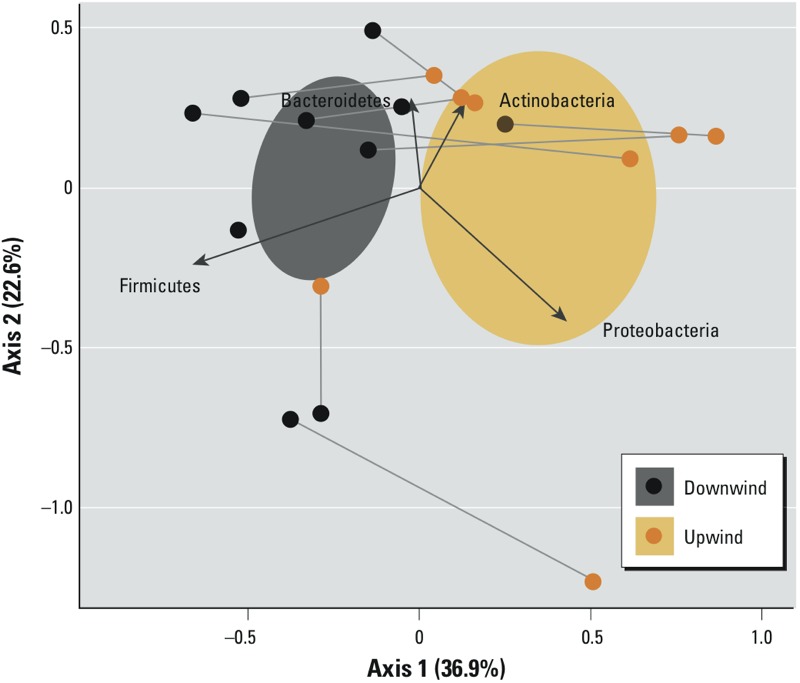
Biplot illustrating Principal Coordinates Analysis (PCoA) of microbial assemblages in airborne PM samples collected upwind and downwind of feed yards located in the Southern High Plains (USA). Each data point represents a sample, and samples from the same feed yard are linked together via a gray line. Two upwind samples were removed from the analysis because of an insufficient number of quality sequencing reads. The proximity of points in this biplot is an approximation of the similarity of samples with respect to their microbiome composition; similarities were measured using the weighted UniFrac measure, which quantifies the phylogenetic distances among samples. Ovals represent the 95% confidence ellipse around the group (upwind vs. downwind) centroids (i.e., multivariate means), and the arrows represent the correlation of individual phyla with PCoA axes. The percent of variability in UniFrac distances accounted for by each axis is indicated.

*Multivariate assessment of microbial communities*. We used UniFrac distance measures and PCoA to assess overall microbial community composition between PM samples collected upwind and downwind of feed yards (Figure 2). Microbial communities in PM samples collected downwind of feed yards tended to be more similar to one another than upwind samples, as depicted by 95% confidence intervals. Bacterial community composition differed significantly between downwind and upwind samples (*p* = 0.005).

**Figure 3:**
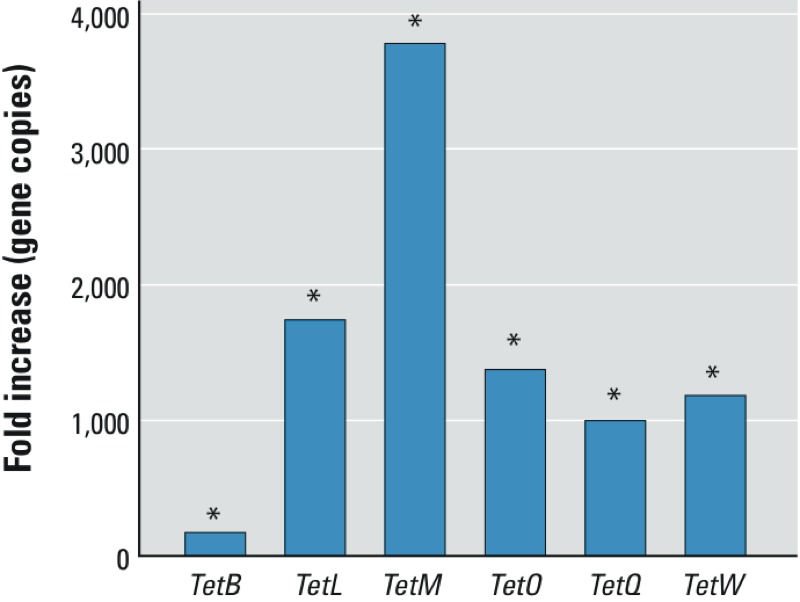
Mean fold increase in abundance of tetracycline resistance genes (*TetB, TetL, TetM, TetO, TetQ, *and* TetW*) in PM collected immediately downwind of feed yards relative to samples collected upwind (*n* = 10/group). All six targeted tetracycline resistance genes were significantly more abundant in PM downwind of feed yards than upwind, with fold change values ranging from 100 to 1,000. Resistance gene *TetM* had the highest fold increase in abundance between PM collected downwind and upwind. *p < 0.002.

*Abundance of antibiotic resistance genes*. Abundances of six targeted tetracycline resistance genes were significantly (all *p* < 0.002) more abundant in PM collected downwind of feed yards compared with those collected upwind (Figure 3). TetQ and TetW were most prevalent across all feed yards (see Supplemental Material, Figure S7). We observed no significant correlation between tetracycline concentrations in PM and tetracycline resistance gene abundance.

## Discussion

To our knowledge, this study is among the first to detect and quantify antibiotics and antibiotic resistance genes and characterize microbial communities associated with airborne PM emitted from beef cattle feed yards. Yet TSP samples collected at feed yard boundaries may not adequately represent particulates blown downwind from the source or those occurring at higher altitudes (Bonifacio et al. 2012; Hiranuma et al. 2011; McGinn et al. 2010). Coarse particles, and those with aerodynamic equivalent diameters > 10 μm tend to settle more quickly than smaller particles. Thus, larger particles may affect areas adjacent to feed yards to a greater extent than smaller (fine) particles. Hiranuma et al. (2011) examined dissipation of PM downwind of a feed yard in the Texas Panhandle to a distance of 3.5 km, and found that PM_10_ concentrations at 3.5 km were approximately 8.5% of the concentrations recorded at the feed yard boundary. However, fine mode particle concentrations declined < 1% at 3.5 km, leading the authors to note that even though fine mode particles contributed less to total PM mass, they would be expected to disseminate over a much broader area. Purdy et al. (2007) also attributed lingering suspensions of feed yard dust in air to small geometric mean sizes of particles occurring within a range of 0.655–0.714 μm.

Feed yards generate significant masses of PM daily. There have been numerous attempts to estimate daily PM_10_ emission factors for beef cattle feed yards, and the range (4.6–127 g/animal/day) of estimates clearly illustrates the variability in both estimation techniques and in the measure itself (McGinn et al. 2010; Parnell 1994). Recently, Bonifacio et al. (2012) estimated emission factors for two feed yards in Kansas to be 27 and 30 g/animal/day. Assuming an average emission rate of 28.5 g PM_10_/animal/day, then 234,840 kg of PM_10_ would have arisen from the 8.24 million cattle on feed yards located in Texas, Oklahoma, Kansas, Nebraska, and Colorado each day during March 2014 (NASS 2014). Sweeten et al. (1998) suggested that PM_2.5_ (particulates ≤ 2.5 μm in aerodynamic diameter that can be inhaled deeply into lungs) represented only about 5% of TSP. Data from a separate study in our laboratory, which examined anabolic steroids associated with PM from feed yards, indicated that 9.17% of PM_10_ collected from feed yards had an AED of ≤ 2.5 μm (Blackwell BR, Wooten KJ, Buser MD, Cobb GP, Johnson BJ, Smith PN, unpublished data). Given this information, we can predict that the mass of PM_2.5_ arising from feed yards in these five states alone would exceed 21,000 kg/day. Assuming that fine particulates from feed yards contain similar concentrations of antibiotics, microbes, and antibiotic resistance genes as quantified in TSP samples collected at feed yard boundaries, and that antibiotics and microbes associated with PM are not destroyed during atmospheric transport, this mass of PM_2.5_ released daily into the air may have significant, far-reaching implications for the spread of antibiotic resistance.

Because the antibiotics quantified in the present study have the potential to be broadly distributed in the environment, it is important to consider their environmental fate and half-lives. Reported half-lives of tetracycline antibiotics in soil and soil-slurry mixes range from 30 to 180 days, whereas half-lives in water are 15–30 days (Kühne et al. 2000). Monensin can remain in manure for > 70 days (Donoho 1984), but tylosin degrades within 30 days (Loke et al. 2000). However, tetracycline compounds and tylosin bind more tightly to soil particles than does monensin and likely also remain tightly bound to airborne particulates (Sarmah et al. 2006). Given the half-lives associated with antibiotics we identified in the present study, it is feasible that these antibiotics remain active during aerial transport and after deposition onto soil, water, or other surfaces for days to weeks.

The concentrations of antibiotics found in airborne PM in the present study were within the ranges of antibiotic concentrations reported in PM collected inside large-scale swine production houses (Hamscher et al. 2003). Swine production facilities are typically fully enclosed, which confines PM and leads to much higher indoor air PM concentrations. Beef cattle feed yards are open-air facilities, which facilitates environmental dispersal of PM via wind. In addition, the antibiotic concentrations we observed in this study are similar to those reported in cattle manure and soils adjacent to beef cattle production facilities (Aust et al. 2008; Christian et al. 2003) and those reported in swine manure (Zhu et al. 2013), suggesting a correlation between antibiotic use and resulting airborne PM-associated veterinary antibiotics. Detections of antibiotics upwind of feed yards were below limits of quantitation, but antibiotic presence upwind indicates that perhaps nonuniform dispersal of PM occurs. In many instances, feed yards used for sample collection in the present study were located in the vicinity of other feed yards. Thus, we cannot discount the possibility that antibiotic residues, microbes, and resistance genes in our samples may have traveled aloft from distant upwind feed yards or originated from the same feed yard previously and returned as a result of subsequent shifts in wind direction. Although our sampling efforts were designed to maximize dust collection while minimizing sampling time, the conditions under which we sampled were typical of those that occur each day at feed yards. Therefore, our samples are representative of those that occur within the season and time periods at feed yards across our study area, but are likely different (i.e., lower dust, bacterial, antibiotic, and ABR gene sequence concentration) from those occurring at other times and during unstable weather.

Bacterial community composition and relative abundance of phyla were similar to those previously reported for cattle fecal matter (Ouwerkerk and Klieve 2001; Rice et al. 2012). In particular, Firmicutes and Bacteroidetes dominated downwind PM samples as expected because they are the two most abundant phyla in cattle fecal matter (Rice et al. 2012). Furthermore, microbial community composition of PM collected immediately downwind of feed yards was distinct from PM collected immediately upwind, confirming the input of feeding operations to bacterial assemblages in downwind environments (Figure 2). Species richness of PM samples evaluated in this study was similar to individual ambient urban air samples (Franzetti et al. 2010); however, it is noteworthy that the OTUs observed in upwind PM tended to be highly variable across the 10 feed yards, whereas OTUs observed downwind of feed yards were substantially less so. This illustrates the degree of bacterial input from feed yards to downwind PM, as well as the variability of activities and sources upwind of feed yards that contribute to the PM bacterial composition.

The present study demonstrates the potential for antibiotics and bacteria to be transported from beef cattle feed yards into the environment by wind. Thus, it is reasonable to consider how far microbes may be transported from these sources, and whether they remain viable after aerial transport. Our study was not designed to address these questions directly, rather it was intended to quantify airborne antibiotics and changes in microbial community composition, as well as to identify antibiotic resistance genes derived from a potential source. Nonetheless, other researchers have documented the potential for dust and associated microorganisms to spread over great distances. For example, trans-Atlantic movement of dust from Africa to Florida, a distance of > 6,500 km, has been reported. Griffin (2007) reported that 99% of those foreign particulates were between 0.3 and 1.0 μm. Air samples collected from multiple aircraft over San Antonio, Texas, in 1965 revealed a stable microbiological population at high altitudes (3,127 m), and indicated that microbial abundance was heavily influenced by temperature inversions and frontal activity (surface and atmospheric turbulence) (Fulton 1966a, 1966b). Jones and Harrison (2004) estimated that > 25% of total airborne particulates occurring in the atmosphere above land masses may consist of microorganisms and organic matter.

Atmospherically suspended microbes are susceptible to a variety of meteorological factors, including ultraviolet light, temperature extremes, and limited moisture. Cole et al. (2008) suggested that the reason gram-negative pathogenic bacteria could not be cultured from open-air feed yard samples [as reported by Purdy et al. (2007) and Wilson et al. (2002)] was because they were quickly killed by irradiation and desiccation. However, these two studies reported successful culture of numerous other microbial genera from airborne particulates. Griffin (2007) hypothesized that upper-altitude dust may attenuate the antimicrobial effect of solar irradiance among bioaerosols suspended at lower altitudes during dust transport. At least one incident of transcontinental transmission of meningococcal meningitis via a dust cloud has been reported (Griffin et al. 2001). Griffin (2007) noted that long-range movement and survival of microbes (some of which are pathogenic) in the atmosphere should be expected based on their ubiquity and adaptability, and that dust likely plays a significant role in biogeographical distribution of pathogenic and nonpathogenic species. However, it is likely that degradation kinetics of antibiotics and ABR gene sequences and survivability of bacteria would be different under conditions with greater solar radiation and temperature.

Genes encoding resistance to tetracycline and sulfonamide antibiotics have been detected in soils adjacent to swine production facilities (Wu et al. 2010), in wastewater lagoons near beef cattle feeding operations (Peak et al. 2007), and in surface waters within a watershed dominated by agricultural facilities (Pei et al. 2006). In order to compare resistance gene concentrations in our study with those of other studies, we normalized gene concentrations to units of gene copies per 16S copies. After normalizing data, we found that tetracycline resistance gene concentrations in lagoon water (Peak et al. 2007) and surface waters (Pei et al. 2006) were lower by several orders of magnitude than those quantified in PM in the present study, indicating that genes encoding resistance can indeed be more prevalent in PM (on a normalized scale) than in other environmental matrices. Interestingly, we detected resistance genes in PM collected upwind of feed yards, albeit at significantly lower concentrations; this indicates the potential for dispersal of feed yard–associated PM counter to prevailing winds on less windy days, or from distant upwind feed yards.

PM-associated antibiotic resistance genes from beef cattle feed yards are of potential concern because of the possibility of lateral gene transfer among the bacterial community (Chee-Sanford et al. 2009). Once in the environment, resistance genes may be transferred between bacteria across a variety of environmental matrices, from soil to PM or PM to surface waters, for example. Although many of the veterinary antibiotics approved for use in beef cattle production are not intended for human use, the potential for resistance to multiple antibiotics within the same class or different classes, including those intended for human use, has been documented (Chee-Sanford et al. 2009). Resistance genes, once in the environment, could be transferred to both nonpathogenic and pathogenic bacteria, which is especially important to consider when taking into account the significant number of wind events that occur across the central United States and specifically in the Southern High Plains, where this research was conducted. Large wind events are capable of global-scale transport and dissemination of PM and microbes.

## Conclusion

PM generated at beef cattle feed yards contains distinct communities of bacteria, antibiotics, and antibiotic resistance gene sequences. Thus, there is significant potential for widespread distribution of antibiotics, bacteria, and genetic material that encodes antibiotic resistance via airborne PM as a result of the large mass of fine particles released daily from beef cattle feed yards in the Central Plains of the United States. Dispersal of PM is facilitated by significant wind energy potentials and frequent wind events in this region. Thus, it follows that feedlot-derived microbes, including those possessing antibiotic resistance, can be transported to new locations where they may occupy new niches (Griffin 2007).

## Supplemental Material

(4.2 MB) PDF
