# Supplementary material for: Antibiotics, Bacteria, and Antibiotic Resistance Genes: Aerial Transport from Cattle Feed Yards via Particulate Matter
Source: Environ Health Perspect. 2015 Jan 22;123(4):337–43. doi: 10.1289/ehp.1408555 (PMC4383574; doi:10.1289/ehp.1408555)
Supplement: (4.2 MB) PDF [file ehp.1408555.s001.508.pdf]

Environmental Health Perspectives doi:10.1289/ehp.1408555

Supplemental Material

Antibiotics, Bacteria, and Antibiotic Resistance Genes: Aerial Transport from Cattle Feed Yards via Particulate Matter

Andrew D. McEachran, Brett R. Blackwell, J. Delton Hanson, Kimberly J. Wooten, Gregory D. Mayer, Stephen B. Cox, and Philip N. Smith

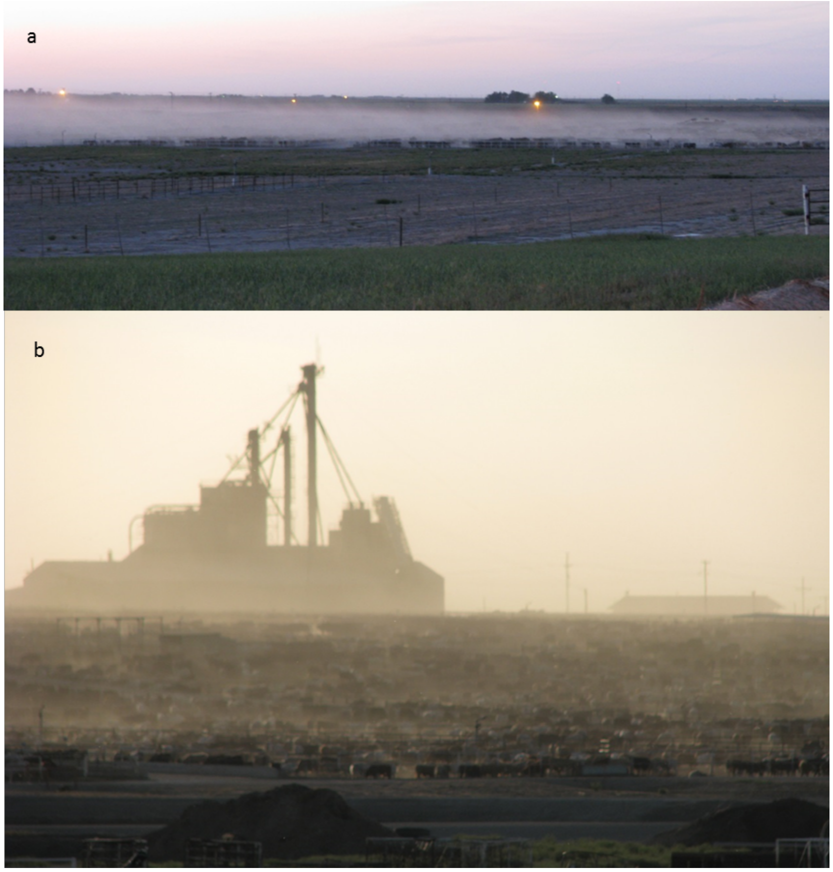

Figure S1. Photographs of evening dust peaks at beef cattle feedyards. Climatic conditions together with feedlot cattle behavioral patterns largely dictate daily suspension of particulate matter above feedyards, a phenomenon that is referred to as the evening dust peak. Relative humidity and soil moisture levels in this region are typically highest in the early morning hours and decline throughout the day. As a consequence, feedyard pen floor material, which consists primarily of urine and fecal material, becomes dry and brittle. Cattle activity and movement increase in late afternoon and early evening, which results in pulverization and subsequent aerosolization of dried pen floor material. Wind speeds and temperatures are generally lowest early in the day, increasing throughout the afternoon. Temperature-driven air inversions also facilitate suspension of particulate matter into air above feedlots. Photo credit: Brett R. Blackwell.

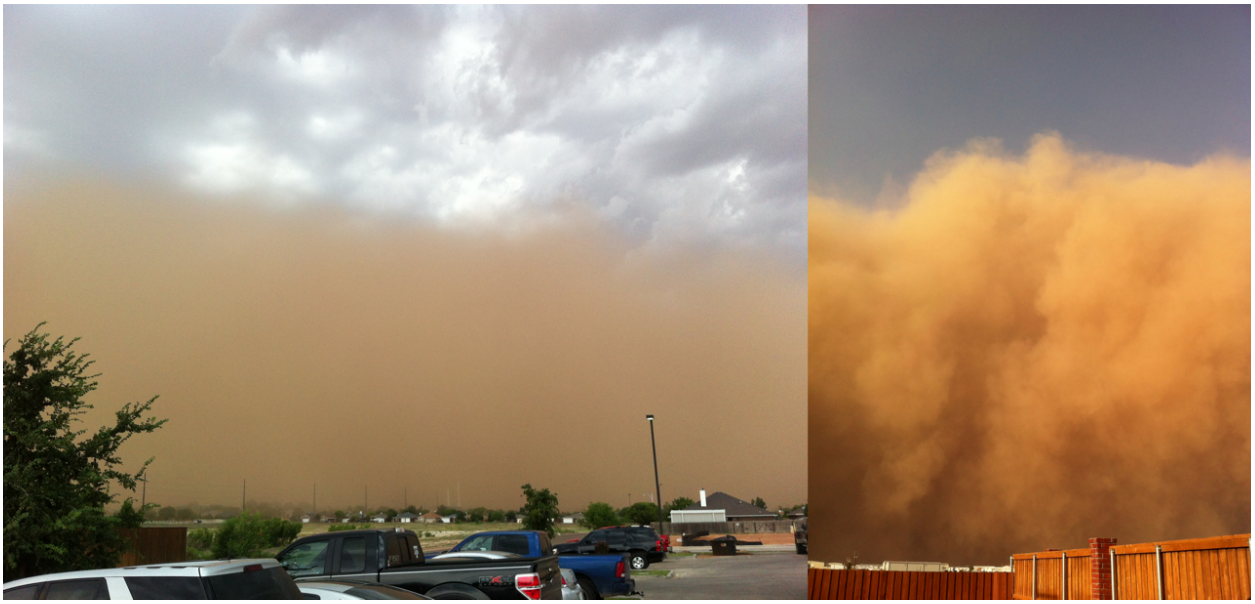

Figure S2. Photographs of two distinct wind and dust events in the Southern High Plains, USA. Photo Credit: Gregory D. Mayer.

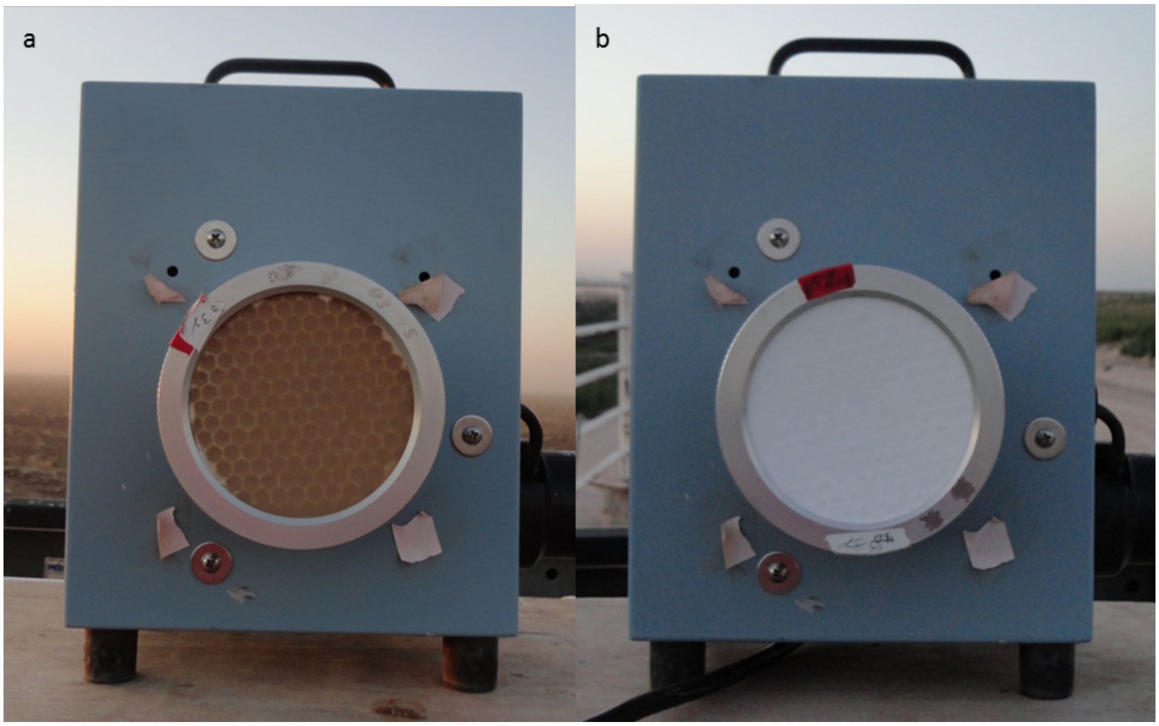

Figure S3. Photographs of total suspended particulate (TSP) samples collected downwind (a), and upwind(b) of feedyard boundaries. Photo credit: Kimblery J. Wooten.

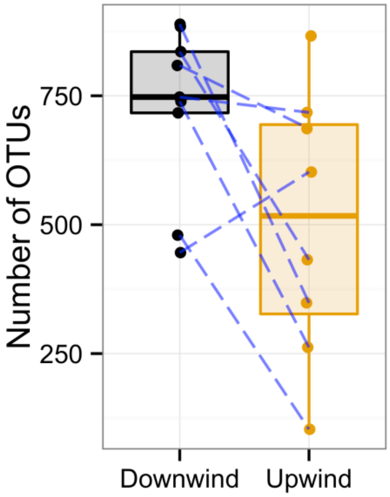

Figure S4. Bacterial richness in airborne PM samples collected upwind and downwind of feedyards located in the Southern High Plains, USA. A boxplot of the number of OTUs per sample is shown. Two upwind samples were removed from analysis because of an insufficient number of quality sequencing reads. Samples from the same feedyard are linked via a light blue line, and to calculate the number of OTUs, the number of sequences per sample was standardized at 2317 using data from rarefaction curves. The median number of OTUs downwind is significantly higher than the number found in the upwind samples.

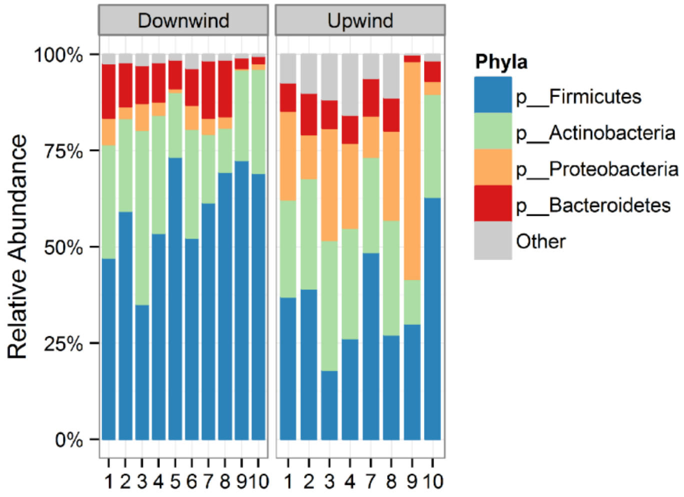

Figure S5. Relative abundance of dominant phyla in airborne PM samples collected upwind and downwind of feedyards located in the Southern High Plains, USA. RDP classifiers are denoted for the four most abundant phyla. Two upwind samples were removed from analysis because of an insufficient number of quality sequencing reads. Firmicutes is the most abundant in downwind samples while Firmicutes and Actinobacteria are similarly abundant in upwind samples.

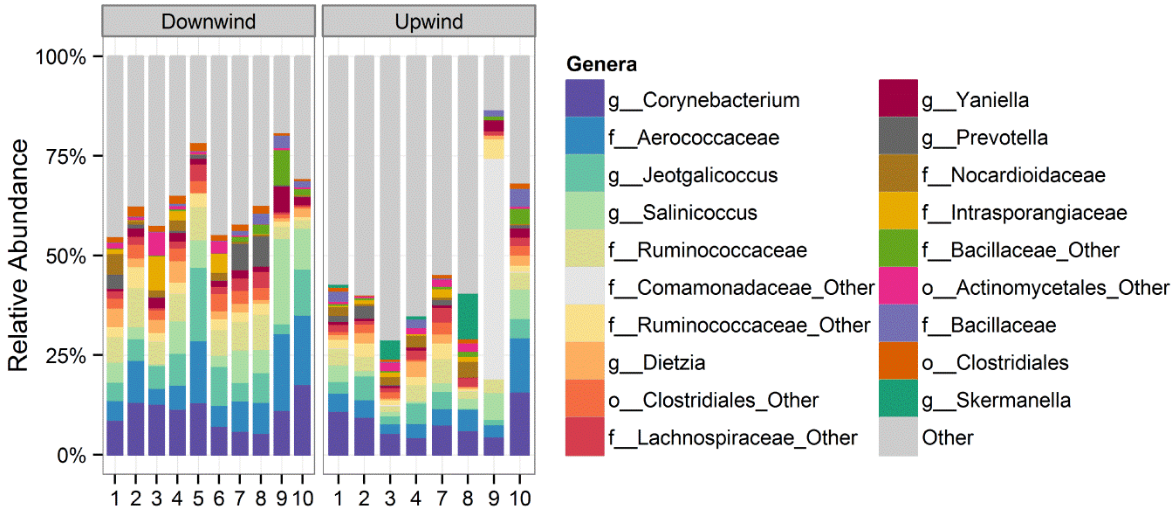

Figure S6. Relative abundance of dominant genera in airborne PM samples collected upwind and downwind of feedyards located in the Southern High Plains, USA. Using the RDP classifier, a group of sequences that cannot confidently be assigned to one taxonomic level are labeled by the next highest level and "_Other". Thus, sequences that cannot be confidently assigned to a genus level are denoted by the family name and "_Other". Other sequences may have a confident match to a reference sequence that is not defined below a certain taxonomic level. These are denoted simply at the family (i.e., f__) or order (i.e., o__) level, depending on what was contained in the reference taxonomy. Two upwind samples were removed from analysis because of an insufficient number of quality sequencing reads.

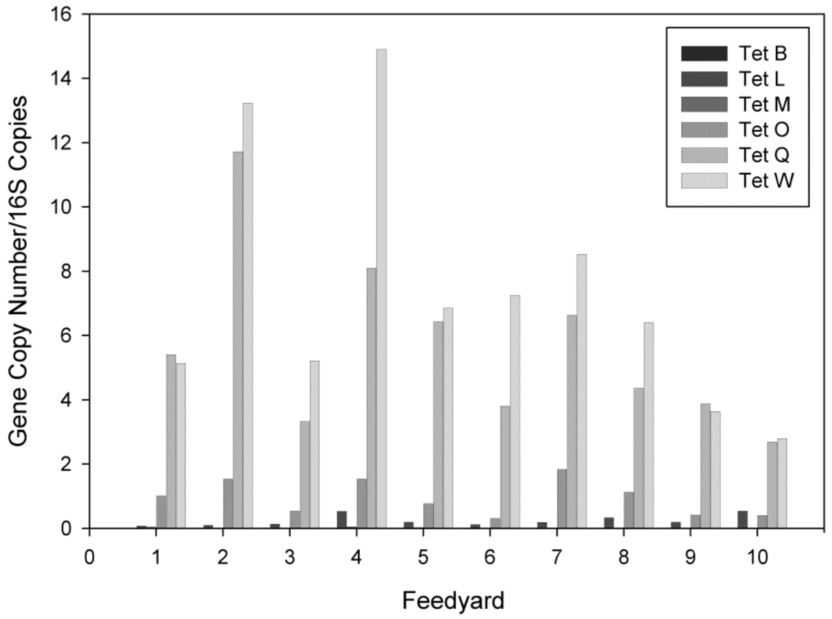

Figure S7. Resistance gene abundance (in gene copies per 16S gene copies) of six targeted tetracycline resistance genes in PM collected immediately downwind of feedyards (n=10), normalized for total copies of 16S genes. Tet Q and Tet W were consistently elevated in abundance across all feedyards.

Table S1. Mobile phase gradient for separation of target analytes via liquid chromatography.

Time	Solvent A (%)	Solvent (B%)
0.00	95	5
6.50	5	95
7.00	5	95
7.50	0	100
9.50	0	100
9.60	95	5
15.00	95	5

Solvent A: water with 0.1% formic acid; Solvent B: acetonitrile with 0.1% formic acid.

Table S2. MS-MS Parameters for ion transitions of all targeted antibiotics plus the internal standard simetone.

Compound	Precursor ion $[M+H]^+$	Product ion 1 (Collision Energy)	Product ion 2 (Collision Energy)	Tube Lens (V)
Tylosin	916.419	173.800 (36)	772.200 (27)	112
Monensin	693.372	461.100 (51)	675.200 (34)	104
Tetracycline	445.100	153.952 (26)	410.013 (18)	71
Chlortetracycline	479.100	444.027 (21)	461.967 (17)	80
Oxytetracycline	461.100	200.897 (34)	426.060 (19)	82
Sulfamethazine	279.029	124.085 (26)	185.952 (17)	66
Methicillin	381.193	165.072 (19)	222.061 (15)	76
Enrofloxacin	360.262	316.200 (18)	342.182 (20)	96
Erythromycin	738.52	161.900 (30)	580.200 (18)	77
Simatone	198.108	68.2 (35)	100.100 (26)	54

Table S3. Primer sets used for tetracycline resistance gene quantification.

Resistance gene	Forward primer	Reverse primer
tet(B)	ACACTCAGTATTCCAAGCCTTTG	GATAGACATCACTCCCTGTAATGC
tet(L)	GGTTTTGAACGTCTCATTACCTGAT	CCAATGGAAAAGGTTAACATAAAGG
tet(M)	GGTTTCTCTTGGATACTTAAATCAATC	CCAACCATAYAATCCTTGTTCRC
tet(O)	AAGAAAACAGGAGATTCCAAAACG	CGAGTCCCCAGATTGTTTTTAGC
tet(Q)	AGGTGCTGAACCTTGTTTGATTC	GGCCGGACGGAGGATTT
tet(W)	GCAGAGCGTGGTTCAGTCT	GACACCGTCTGCTTGATGATAAT

Based on Peak, et al. (2007) and Smith, et al. (2004).

Table S4. Sampling weather conditions at all feedyards (n=10).

Feedlot #	Sampling month	Temp C	Wind speed (m/s)	Relative humidity (%)	Precipitation (cm)
1	11	19	0.9	23	0
2	11	10	3.6	27	0
3	11	17	1.3	26	0
4	12	15	3.1	29	0
5	12	12	0.9	22	0
6	12	16	2.7	29	0
7	12	16	4.0	18	0
8	12	16	4.0	18	0
9	8	32	6.7	31	0
10	8	31	2.7	38	0

Table S5. Mean (and standard error) of PM mass collected upwind and downwind of each feedyard (n=10).

Feedlot ID	Downwind mass (mg)	Upwind mass (mg)
1	87.95 (6.33)	9.00 (3.10)
2	12.98 (1.38)	0.27 (0.26)
3	96.78 (8.18)	1.57 (0.32)
4	92.60 (1.75)	1.45 (1.80)
5	204.3 (20.65)	10.65 (2.13)
6	72.50 (3.89)	0.03 (0.26)
7	179.9 (7.77)	0.40 (0.41)
8	185.8 (12.28)	9.85 (1.08)
9	326.4 (21.17)	2.25 (0.22)
10	58.70 (5.97)	3.03 (0.74)

References

Peak N, Knapp CW, Yang RK, Hanfelt MM, Smith MS, Aga DS, et al. 2007. Abundance of six tetracycline resistance genes in wastewater lagoons at cattle feedlots with different antibiotic use strategies. Environ Microbiol 9:143-151.

Smith MS, Yang RK, Knapp CW, Niu Y, Peak N, Hanfelt MM, Galland JC, Graham DW. 2004. Quantification of tetracycline resistance genes in feedlot lagoons by real-time PCR. Appl Environ Microbiol 70: 7372-7277.
